# Case Report: Benign colonic schwannoma with restricted diffusion mimicking malignancy

**DOI:** 10.3389/fonc.2026.1895724

**Published:** 2026-07-09

**Authors:** Hao Zhu, Xiuya Cen, Chunchun Li, Deen Shi, Yongchou Li, Shitian Bao, Wei Weng

**Affiliations:** 1 Department of Radiology, The Third Affiliated Hospital of Wenzhou Medical University, Wenzhou, Zhejiang, China; 2 Department of Pathology, The Third Affiliated Hospital of Wenzhou Medical University, Wenzhou, Zhejiang, China

**Keywords:** colonic schwannoma, gastrointestinal schwannoma, malignancy mimic, mesocolic lymphadenopathy, restricted diffusion

## Abstract

**Introduction:**

Colonic schwannoma is a rare gastrointestinal mesenchymal tumor that is usually benign but may be difficult to distinguish from malignancy before surgery. This case is notable because a benign colonic schwannoma showed both pronounced restricted diffusion on MRI and prominent mesocolic lymphadenopathy, creating a strong radiologic impression of malignancy.

**Case presentation:**

A 43-year-old woman presented with a 2-day history of abdominal pain accompanied by altered bowel habits. Colonoscopy revealed a large spherical submucosal mass in the transverse colon. Biopsy suggested a neurogenic tumor favoring schwannoma, but malignancy could not be excluded because of limited sampling. Contrast-enhanced CT showed a large colonic mass with heterogeneous internal density, focal cystic change, irregular serosal appearance, and multiple enlarged mesocolic lymph nodes. MRI demonstrated marked restricted diffusion, with hyperintensity on diffusion-weighted imaging and hypointensity on ADC maps.

**Diagnosis, interventions, and outcomes:**

The lesion was clinically suspected to be a malignant colonic tumor. The patient underwent laparoscopic adhesiolysis and left hemicolectomy. Postoperative histopathology unexpectedly confirmed a benign gastrointestinal schwannoma. All 34 harvested mesocolic lymph nodes showed reactive hyperplasia without metastasis. The patient recovered after surgery and was discharged following supportive treatment.

**Conclusion:**

Benign colonic schwannoma can closely mimic malignancy when restricted diffusion and prominent regional lymphadenopathy are present. This single case underscores that such features are not specific for malignancy; because imaging and biopsy could not exclude a malignant tumor, oncologic resection remained appropriate, and the definitive diagnosis still depended on histopathology and immunohistochemistry.

## Introduction

Schwannomas are benign mesenchymal tumors arising from the Schwann cells of peripheral nerve sheaths. Although the gastrointestinal tract is an uncommon site, schwannomas may occur throughout it, with the stomach being the most frequent location and the colon and rectum being rare (1–3). Colorectal schwannomas usually present as slow-growing submucosal or mural lesions and are almost always benign, with an excellent prognosis after complete resection (1–3). Because their endoscopic and cross-sectional imaging appearances overlap with those of other mural spindle-cell tumors, particularly gastrointestinal stromal tumor (GIST), a definitive diagnosis is frequently established only after pathological and immunohistochemical examination of the resected specimen (2–4).

A particular diagnostic difficulty arises when a benign colorectal schwannoma displays imaging features that are conventionally regarded as signs of malignancy, such as large size, heterogeneous enhancement and regional lymph-node enlargement (4–6). Diffusion-weighted imaging (DWI) is increasingly used in the assessment of colorectal tumors because malignant lesions tend to show restricted diffusion related to high cellularity (7, 8); however, restricted diffusion is not specific for malignancy and may also occur in highly cellular benign tumors (9).

Herein, we report a benign, cellular colonic schwannoma that simultaneously demonstrated marked restricted diffusion on MRI and prominent mesocolic lymphadenopathy, producing a strong preoperative impression of malignancy. We describe the clinical, imaging and pathological findings, discuss the mechanisms underlying these pseudo-malignant features and review the relevant literature, with the aim of alerting radiologists and clinicians to this uncommon diagnostic pitfall.

## Case presentation

A 43-year-old woman was admitted with a 2-day history of abdominal pain accompanied by altered bowel habits. On admission, abdominal examination showed mild upper abdominal tenderness without rebound tenderness or a palpable mass. Fecal occult blood testing was positive, whereas serum tumor markers, including AFP, CEA, CA125, CA153, CA19-9, and CA724, were not elevated. Colonoscopy demonstrated a large, spherical submucosal mass approximately 55–60 cm from the anal verge, covered with thick whitish exudate (**Figure 1**). Biopsy showed chronic active inflammation with stromal spindle-cell aggregates. Immunohistochemistry suggested a neurogenic tumor, favoring schwannoma, with diffuse strong S-100 positivity and low Ki-67 expression. However, because of the limited biopsy sample, malignancy could not be confidently excluded. Contrast-enhanced CT revealed a slightly hypodense, round mass arising from the transverse colon near the splenic flexure, with mild-to-moderate enhancement, heterogeneous internal density, focal cystic change, irregular serosal surface, and multiple enlarged mesocolic lymph nodes (**Figure 2**). MRI further characterized the lesion as a colonic wall mass with heterogeneous signal intensity and pronounced restricted diffusion, appearing hyperintense on DWI and hypointense on ADC maps (**Figure 3**). On region-of-interest measurement, the mean apparent diffusion coefficient (ADC) of the mass was low, approximately 0.64 × 10^-3^ mm²/s (642.67 × 10^-6^ mm²/s; region-of-interest area, 19.63 mm²), consistent with markedly restricted diffusion. Taken together, these findings strongly suggested a malignant colonic tumor despite the biopsy result. After preoperative evaluation, the patient underwent laparoscopic adhesiolysis and left hemicolectomy. Intraoperatively, a firm mass measuring approximately 7 × 6 × 5 cm was identified in the transverse colon near the splenic flexure, with serosal involvement. Multiple enlarged lymph nodes were observed at the roots of the inferior mesenteric and middle colic arteries. No obvious peritoneal, splenic, or pelvic metastases were found. Postoperative histopathology unexpectedly confirmed a benign, cellular gastrointestinal schwannoma, showing spindle cells arranged in intersecting fascicles with lymphoplasmacytic infiltration and focal inflammatory necrosis associated with the lymphoplasmacytic infiltrate, without coagulative tumor-type necrosis (**Figure 4**). Immunohistochemically, the tumor cells were positive for S-100 and NSE and negative for DOG1, CD117, CD34 (positive only in vessels), PDGFRA, h-caldesmon, desmin, smooth-muscle actin, synaptophysin and STAT6; CD99 was positive. The Ki-67 proliferation index was approximately 5%, and phosphohistone-H3 (PHH3) labeling indicated low mitotic activity (<1%). This panel excluded gastrointestinal stromal tumor, smooth-muscle tumor, neuroendocrine tumor and solitary fibrous tumor, and confirmed a benign gastrointestinal schwannoma. All 34 harvested mesocolic lymph nodes showed reactive hyperplasia without metastasis. The patient recovered after surgery and was discharged following continued supportive treatment.

**Figure 1:**
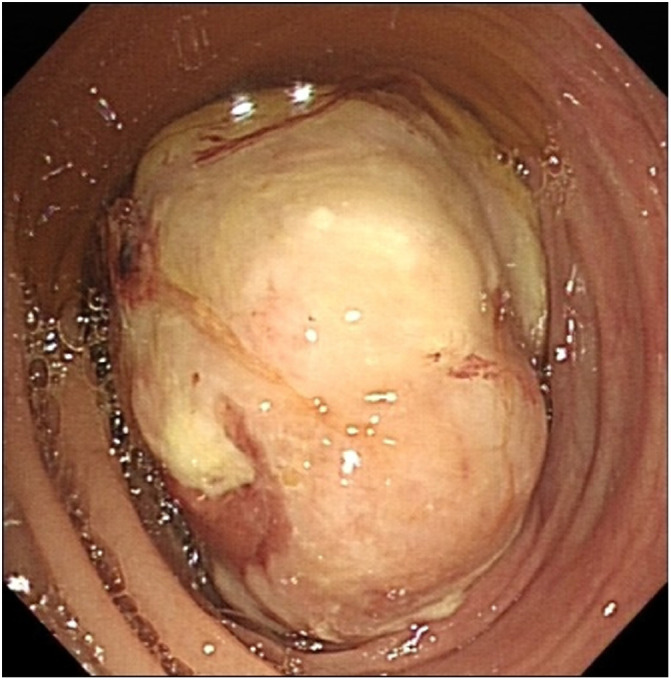
Colonoscopy revealing a large, spherical, submucosal mass covered with a thick, whitish exudate approximately 55–60 cm from the anal verge.

**Figure 2:**
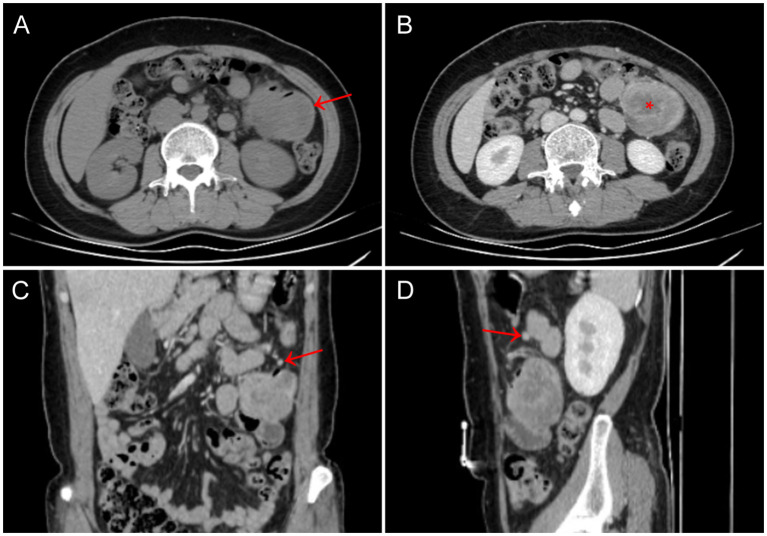
Abdominal CT images of the colonic mass. **(A)** Unenhanced axial image showing a slightly hypodense, round mass arising from the transverse colon near the splenic flexure (arrow). **(B)** Contrast-enhanced axial image demonstrating annular, mild-to-moderate enhancement with heterogeneous internal density and focal cystic changes (asterisk). **(C)** Coronal and **(D)** sagittal contrast-enhanced images revealing intraluminal and extramural growth, an irregular serosal surface, and multiple enlarged mesocolic lymph nodes (arrows).

**Figure 3:**
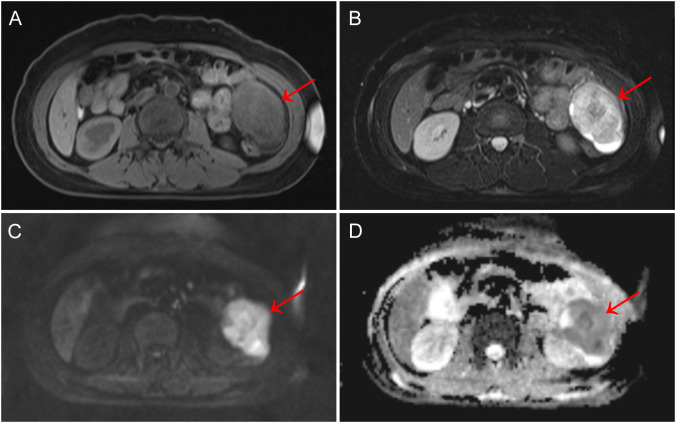
Abdominal MRI of the colonic mass. **(A)** Axial T1-weighted image revealing a slightly hypointense mass (arrow). **(B)** Axial T2-weighted image demonstrating heterogeneous, slight hyperintensity (arrow). **(C)** High-b-value diffusion-weighted imaging (DWI) exhibiting marked hyperintensity (arrow). **(D)** Apparent diffusion coefficient (ADC) map showing hypointensity, confirming restricted diffusion (mean ADC of the mass, approximately 0.64 × 10^-3^ mm²/s)(arrow).

**Figure 4:**
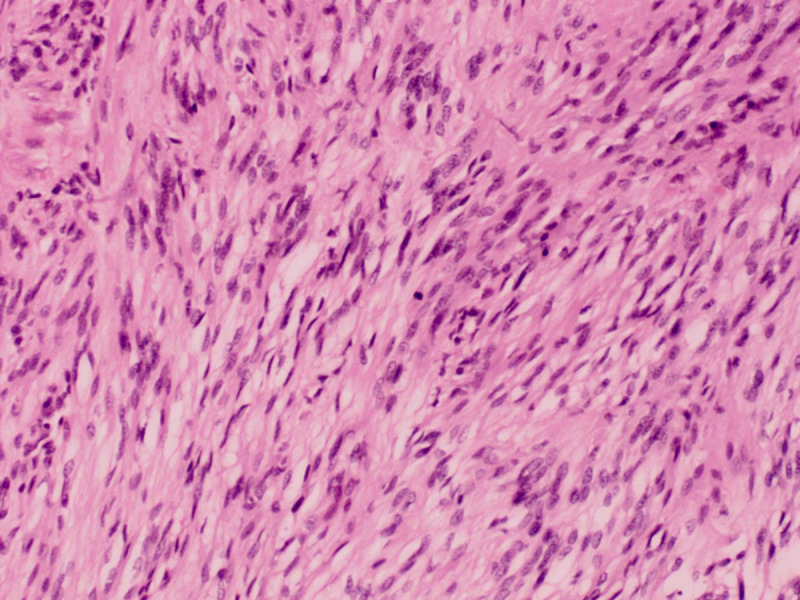
Histopathological examination of the resected specimen showing spindle cells arranged in intersecting fascicles, accompanied by lymphoplasmacytic infiltration and focal inflammatory necrosis (H&E stain).

## Discussion

The present case illustrates an unusual diagnostic pitfall: a benign colonic schwannoma closely mimicking malignancy because of the combined presence of a large colonic mass, marked restricted diffusion, irregular serosal appearance, and prominent mesocolic lymphadenopathy. Colorectal schwannoma is rare, and previous reviews have shown that it is often recognized as a submucosal lesion but definitively diagnosed only after pathological examination of the resected specimen (1–3). In our case, colonoscopic biopsy suggested a neurogenic tumor, but the overall preoperative impression remained malignant because the imaging findings were highly suspicious.

The first deceptive feature was the CT appearance. The mass arose from the transverse colon near the splenic flexure and showed heterogeneous internal density, focal cystic change, irregular serosal surface, and multiple enlarged mesocolic lymph nodes. Kang et al. reported that colorectal schwannomas may show enlarged lymph nodes on CT and that this feature can help distinguish schwannomas from colorectal GISTs in some settings (4). However, when a large colonic mass is accompanied by serosal irregularity and regional lymphadenopathy, the more common clinical interpretation is colonic malignancy. This explains why the imaging findings in our case outweighed the relatively benign tendency suggested by biopsy.

The second and most striking misleading feature was the regional lymphadenopathy. In colorectal cancer, enlarged regional lymph nodes usually raise concern for nodal metastasis. However, gastrointestinal schwannomas may be associated with reactive lymphadenopathy. Bae et al. demonstrated that regional lymphadenopathy in gastrointestinal schwannomas correlates with a peritumoral lymphoid cuff (5). Shu et al. also reported reactive hyperplasia in lymph nodes surrounding intestinal schwannomas (6). These observations are highly relevant to our case, in which all 34 harvested mesocolic lymph nodes showed reactive hyperplasia without metastasis. Therefore, the striking lymphadenopathy in this case should be interpreted as a pseudo-malignant feature rather than true malignant spread.

The third deceptive feature was the marked restricted diffusion on MRI. DWI is widely used in colorectal cancer imaging because it improves tumor conspicuity and reflects restricted water mobility, often related to high cellularity (7, 8). In our case, the lesion was hyperintense on DWI and hypointense on ADC maps, a pattern that strongly reinforced the suspicion of malignancy. Nevertheless, restricted diffusion is not specific for malignant tumors. ADC values are influenced by tissue cellularity and microstructure, and benign lesions with high cellularity may also show low ADC values (9). Moreover, MRI-based nodal assessment in rectal cancer has only moderate accuracy, and adding DWI does not necessarily improve nodal staging performance (10, 11). It should be noted that these data are derived from rectal rather than colonic cancer and must therefore be extrapolated to the colon with caution; nonetheless, they indicate that diffusion characteristics and nodal size are imperfect predictors of malignancy. Thus, the present case highlights that restricted diffusion should be interpreted cautiously, especially when pathological and immunohistochemical findings suggest a benign mesenchymal tumor.

Another important differential diagnosis in this case was GIST. Colonic schwannoma and GIST can both present as submucosal or mural spindle-cell tumors, and imaging findings may overlap. GISTs may show heterogeneous enhancement, cystic change, and diffusion restriction, particularly in larger lesions (12). Therefore, imaging alone cannot reliably distinguish these entities. Immunohistochemistry remains essential: DOG1 and CD117 are key markers for GIST diagnosis (13, 14), whereas gastrointestinal schwannomas are typically S-100 positive and negative for DOG1 and CD117 (2, 3, 6). In our case, this immunohistochemical profile supported schwannoma rather than GIST. Beyond GIST, the differential diagnosis of a colonic spindle-cell tumor also includes leiomyoma and neurofibroma. Leiomyomas are typically positive for smooth-muscle actin and desmin and negative for S-100, whereas neurofibromas, although also S-100 positive, usually contain a mixed population of Schwann cells, fibroblasts and perineurial cells, are more often associated with neurofibromatosis type 1, and lack the uniformly strong, diffuse S-100 pattern seen in our case. Importantly, no single imaging feature in our patient reliably favored schwannoma over a malignant epithelial tumor or GIST; the only preoperative finding pointing to the correct diagnosis was the S-100-positive endoscopic biopsy, and this was outweighed by the aggressive imaging appearance. Definitive differentiation was therefore possible only after histopathological and immunohistochemical examination of the resected specimen.

The management of this case was influenced by the malignant-appearing imaging features. Although gastrointestinal schwannomas generally have a favorable prognosis and low recurrence risk after complete resection (2, 3), preoperative diagnosis is often uncertain. In our patient, the large tumor size, serosal irregularity, restricted diffusion, and extensive lymphadenopathy made oncologic resection clinically reasonable. Final pathology, however, confirmed benign gastrointestinal schwannoma with reactive lymph nodes. This contrast between aggressive imaging appearance and benign pathology is the central message of the case.

In conclusion, we describe a benign, cellular colonic schwannoma in which marked restricted diffusion, an irregular serosal surface, and prominent mesocolic lymphadenopathy together produced a convincing imaging impression of malignancy. These features—although usually associated with colorectal carcinoma or GIST—can also arise from benign mechanisms, in this case the high cellularity of the tumor and a peritumoral lymphoid reaction. We do not propose that these findings allow schwannoma to be reliably distinguished from malignancy before operation: in our patient, imaging could not exclude a malignant tumor, the endoscopic biopsy was not definitive, and oncologic resection therefore remained an appropriate and prudent decision. Rather, this single case is a reminder that restricted diffusion and regional lymphadenopathy are not specific for malignancy, and that the definitive diagnosis of mesenchymal colonic tumors continues to rest on histopathology and immunohistochemistry. Larger studies would be needed before any change to imaging interpretation or surgical management could be justified.

## Data Availability

The original contributions presented in the study are included in the article. Further inquiries can be directed to the corresponding author.
